# Microstructure and Properties of Aluminum Alloy/Diamond Composite Materials Prepared by Laser Cladding

**DOI:** 10.3390/ma17215280

**Published:** 2024-10-30

**Authors:** Shuhui Huang, Yilin Zhao, Haofeng Xie, Hong Guo, Lijun Peng, Wenjing Zhang

**Affiliations:** 1 State Key Laboratory of Nonferrous Metals and Processes, China GRINM Group Co., Ltd., Beijing 100088, China; hithuang@126.com (S.H.); freewaka@outlook.com (Y.Z.); guohong@grinm.com (H.G.); penglj@grinm.com (L.P.); zhangwenjing@grinm.com (W.Z.); 2 GRIMAT Engineering Institute Co., Ltd., Beijing 101407, China; 3 General Research Institute for Nonferrous Metals, Beijing 100088, China

**Keywords:** laser cladding, aluminum alloy/diamond composite material, tungsten coating, microstructure, thermal and mechanical properties

## Abstract

In this article, AlSi10Mg aluminum alloy was used as the substrate to prepare aluminum alloy/diamond composite materials with laser cladding technology. The effects of the composition and laser power on the microstructure and thermal properties of the composite materials were studied. The results show that the prefabrication of tungsten carbide layer on the diamond surface enhances the wettability of diamond with aluminum alloy and reduces the laser reflection, which ensures the implementability of laser cladding technology for the preparation of aluminum alloy/diamond composites. The laser power and components determine the temperature of the molten pool and thus the state of the organization of the composite material to be formed by cladding. With the increase in the diamond content, the density, specific heat, mechanical properties, and average linear thermal expansion coefficient of the composite material gradually decrease, while the thermal conductivity first increases and then decreases. The thermal conductivity of the aluminum alloy/diamond composite material prepared by laser cladding is 200.68 W/mK, and the linear thermal expansion coefficient is 1.904 × 10^−5^/K, which are superior to those of the matrix AlSi10Mg aluminum alloy.

## 1. Introduction

With the development of semiconductor and microelectronics technology, the power density of electronic devices is becoming higher and higher, leading to a sharp increase in their heat generation. The heat dissipation of high-power-density electronic devices has gradually become a stranglehold problem restricting improvements in their performance. According to statistics released by the United States, over half of all electronic device failures are caused by difficulties in heat dissipation [1,2]. Thermal management materials are used as substrate materials, heat sink materials, and other critical parts of electronic packages. Their main function is to conduct heat and support and protect chips and electronic components. Metal/diamond composite materials have the characteristics of high thermal conductivity and low expansion, and are currently the most suitable heat sink materials for chips. The metals that can form composite materials with diamond mainly include silver, copper, and aluminum. Copper/diamond composite materials and aluminum/diamond composite materials have been industrialized and applied. Copper/diamond composite materials exhibit a better thermal conductivity and thermal expansion performance than aluminum/diamond composite materials. However, from the perspectives of manufacturing difficulty, product scale, density and cost, aluminum/diamond composites are undoubtedly the more attractive option on the market [3]. In 1995, Lawrence Livermore National Laboratory in the United States collaborated with Sun Microsystems and introduced metal/diamond composite materials prepared by vacuum pressure infiltration [4]. Subsequently, Plansee in Austria used gas pressure-assisted infiltration (GPI) to prepare metal/diamond composite materials with a thermal conductivity of 450–550 W/mK and a thermal expansion coefficient of 7.5–8.5 ppm/K from room temperature to 120 °C, which was an advanced product in the international market at that time [4]. Finite element technology and theoretical model calculations have played a significant role in the design of metal diamond composite materials. The Hasselman–Johnson model, which has been tested in practice, is more accurate compared to other models [5,6].

At the beginning of this century, Japanese companies such as Sumitomo Electric, DENKA, and Osaka University also successively mastered the industrial technology of pressure infiltration to prepare metal/diamond composite materials. Their product performance is similar to that of Plansee company’s product, and they are also very competitive in the international market [7]. Around 2007, some research institutions in China, such as the General Research Institute for Nonferrous Metals, Beijing University of Science and Technology, Shanghai Jiao Tong University, Harbin Institute of Technology, Hunan University, etc., began to conduct research on metal/diamond composite materials. The prepared metal/diamond composite materials have a thermal conductivity of 500~800 W/mK and a thermal expansion coefficient of 7.5~8.5 ppm/K from room temperature to 120 °C [8,9].

At present, the preparation methods of metal/diamond composite materials mainly include powder metallurgy and liquid-phase methods. Powder metallurgy methods include hot pressing sintering, discharge plasma sintering, and high-temperature and high-pressure sintering. Liquid phase methods include squeeze casting, pressureless self-infiltration, and gas pressure infiltration [8,9,10,11]. Metal/diamond composite materials prepared by pressure infiltration exhibit a better performance under the same composition, which is currently better than the performance of materials made using older methods for preparing metal/diamond composite materials. Under high temperature and pressure conditions, diamond is tightly wrapped in a liquid aluminum alloy. During the subsequent cooling process, sustained pressure maintains a tight interface between the aluminum alloy and diamond [9].

The shape of the prepared diamond/metal composite blanks should not be too complex, whether it is prepared by powder metallurgy or a liquid-phase method. The specifications, efficiency, and cost of related products cannot meet all market demands. If metal/diamond composite materials with unlimited specifications and shapes can be prepared, even with reduced mechanical and thermal properties, they will still be very attractive products on the market. They can replace metal materials in many applications to improve equipment performance.

Laser cladding forming (LCF) is a new manufacturing technology that combines rapid prototyping with laser cladding surface strengthening. Laser cladding technology can be used to prepare structurally complex parts, gradient materials, and products that are almost unrestricted by size with a near net form [12,13,14].

Researchers have used laser cladding to prepare various metal matrix composite materials. In reference [15], the researchers mixed nano TiB2 with nickel-based high-temperature alloy powder and Al_2_O_3_ powder with titanium aluminum alloy powder, and printed materials using laser cladding technology to improve their high-temperature mechanical properties. Reference [16] reported the preparation of graphite/copper gradient composite materials using laser cladding technology as pantograph slides for high-speed trains, significantly improving the material thermal conductivity and wear resistance. Reference [17] introduced the preparation of TiC/Ti6Al4V composite materials using laser cladding technology. Compared with Ti6Al4V matrix alloy, the strength of TiC/Ti6Al4V composite materials by laser cladding increased, and the toughness was also significantly improved. According to the needs of the application, the addition of reinforcement to the metal matrix can achieve the regulation and exploitation of material properties such as force, heat, electricity and wear resistance. At present, powders used as reinforcements mainly include oxides, carbides, and nitrides, such as Al_2_O_3_, ZrO_2_, Cr_2_O_3_, TiO_2_, WC, TiC, SiC, B_4_C, Cr_3_C_2_, TiN, Si_3_N_4_, etc. These reinforcements can improve the thermal and mechanical properties of the matrix alloy [16,17,18,19,20,21,22].

Diamond, as a special reinforcing material, can be classified into carbides. The thermal conductivity of diamond particles can reach 2000 W/mK, and the coefficient of thermal expansion is only 2 ppm/K. The application of metal/diamond composite materials in the field of thermal management has been described previously. Currently, there are no reports on the preparation of aluminum alloy/diamond composites using laser cladding. This is due to the risk of graphitization transformation of diamond when using diamond as a reinforcing material for laser cladding (the graphitization temperature of diamond is about 1500 °C in vacuum or inert gas). Therefore, only aluminum alloys with low melting points can be selected as the matrix when preparing metal/diamond composites by laser cladding.

In both references [23,24], diamond composite grinding wheels were prepared using laser cladding technology. It can be seen that the most significant problem in the preparation of metal diamond composite materials by laser cladding is the defects at the interface between the metal and diamond. How to reduce the content of defects is the key to improving the performance of composite materials.

The author explores the feasibility of using laser cladding technology to prepare aluminum alloy/diamond composite materials in this article, and focuses on the influence of the laser cladding power and composition on the formability of composite materials. An aluminum alloy/diamond composite with densification surface was prepared, and the microstructure and thermal properties of the composite were characterized, including the density, specific heat, thermal diffusion coefficient, thermal expansion coefficient, and bending strength. The experimental data in this article can provide a reference for further optimization of the preparation of aluminum alloy/diamond composite materials using laser cladding technology.

## 2. Materials and Methods

The raw materials used in the experiment are tungsten-plated diamond and AlSi10Mg alloy powder. The average particle size of diamond is (D50) 100 μm, and the surface is coated with tungsten by magnetron sputtering, with a tungsten plating layer of about 100 nm. The average powder particle size of AlSi10Mg alloy is (D50) 30 μm. AlSi10Mg powder is a common additive manufacturing raw material that can be used in processes such as Selective Laser Melting (SLM), Electron Beam Melting (EBM), Direct Laser Deposition (DLD), powder metallurgy (PM), Injection Molding (MIM), and laser cladding [25]. In this article, diamond and aluminum alloy powders are prepared as composite materials according to the volume fraction ratio.

**Figure 1:**
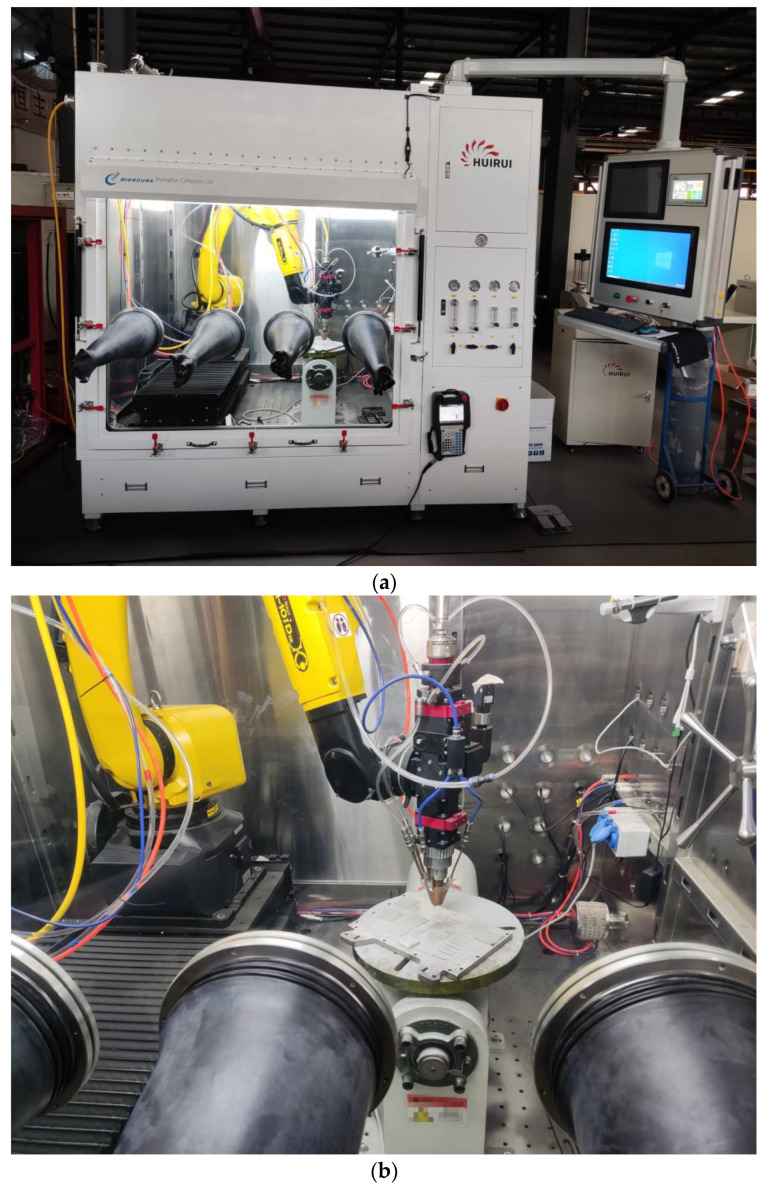
Metal-1006 laser cladding equipment: (**a**) outside, (**b**) inside.

The Metal-1006 equipment developed by Huirui Laser Technology Co., Ltd. of Tianjin, China was used for laser cladding experiments, as shown in Figure 1. This device is equipped with the Ruike RFL-C6000XC laser module of Wuhan, China which has an adjustable laser power within 500–4000 W and a laser wavelength of 1070 ± 10 nm. The powder feeding rate of the equipment can be adjusted to be between 1~400 g/min, and the protective gas is high-purity argon gas. The Yokogawa GM90PS multi-channel thermometer was used to measure the relationship between laser power and spot temperature.

Analysis equipment including PANalytic X-Pert3 Powder equipment of Holland (X-ray diffractometer, XRD), Thermo ESCALAB Xi+equipment of America (X-ray Photoelectron Spectroscopy, XPS), Hitachi S-4800 equipment of Japan (scanning electron microscope, SEM), NETZSCH STA449 synchronous thermal analyzer of Germany, NETZSCH DIL402 thermal expansion analyzer of Germany, and WDW-100 tensile testing machine of China were used in this study.

The standards followed in the testing are as follows: “GB/T 1423-1996 Test Method for Density of Precious Metals and Their Alloys [26]”, “GB/T 4339-2008 Determination of Thermal Expansion Characteristic Parameters of Metallic Materials [27]”, “GB/T 22588-2008 Flash Method for Measuring Thermal Diffusion Coefficient or Thermal Conductivity [28]”, “ASTM E1269-11 Standard Test Method for Determining Specific Heat Capacity by Differential Scanning Calorimetry [29]”, and “GB/T 6569-2006 Test Method for Bending Strength of Fine Ceramics [30]”.

## 3. Results

### 3.1. Surface Treatment of Diamond

Diamond has a strong inertness and is difficult for it to achieve good interfacial bonding with metals under general conditions. Introducing a carbide layer between the diamond and metal can improve the wetting and bonding of the metal to the diamond, thereby achieving the goal of enhancing the overall performance of the composite material. Carbides formed by elements such as boron, titanium, chromium, zirconium, and tungsten have been reported to improve the interface between the diamond and metal [31,32]. The carbides formed using W include W_2_C and WC, which have significantly different thermal conductivities. The thermal conductivity of W_2_C is only 36 W/mK, while the thermal conductivity of WC reaches 120 W/mK.

The use of tungsten-plated diamond to prepare metal diamond composite materials has achieved good results in practice. In references [4,33], aluminum alloy diamond composite materials and copper alloy diamond composite materials were prepared using tungsten-plated diamond, respectively. The thermal conductivity of the composite materials reached 294 W/mK and 720 W/mK.

In this article, the author first uses magnetron sputtering technology to deposit a tungsten layer on the surface of diamond, and then obtains a WC layer through vacuum heat treatment process, providing raw materials for the preparation of the aluminum alloy/diamond composite materials. 

The heat treatment process for the tungsten-plated diamond is as follows: (room temperature → 1150 °C)/60 min → 1150 °C/15 min → (1500 °C → room temperature)/furnace cooling, with a vacuum degree maintained at the level of 10^−3^ Pa during the treatment process.

**Figure 2:**
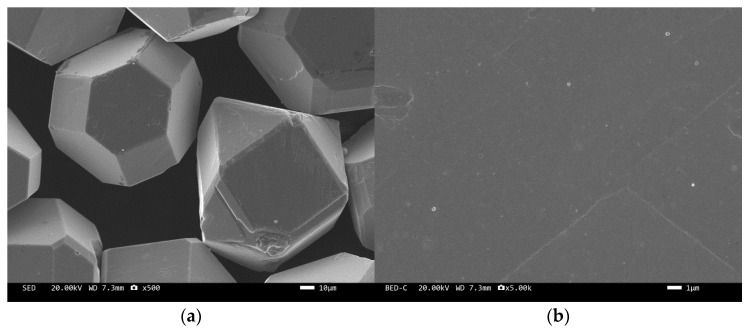
SEM image of tungsten-plated diamond before heat treatment: (**a**) ×500, (**b**) ×5000.

**Figure 3:**
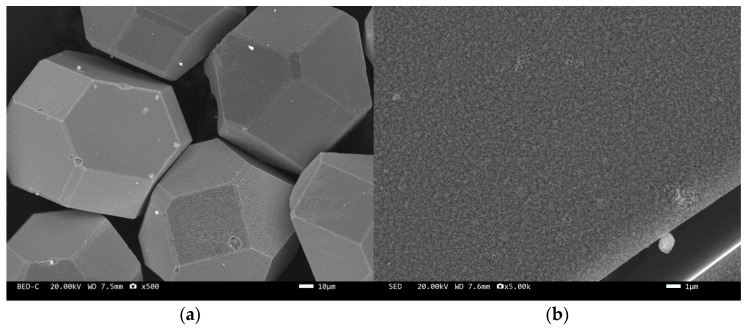
SEM image of tungsten-plated diamond after heat treatment: (**a**) ×500, (**b**) ×5000.

The microstructure of the tungsten-plated diamond before and after the heat treatment is shown in Figure 2 and Figure 3. It can be seen that the coating distribution on the surface of the tungsten-plated diamond particles is very flat and smooth, without any missed plating. The diamond particles are intact and have similar sizes. After the heat treatment, the morphology of the diamond surface coating changed significantly, and the coating was no longer smooth but appeared as fine particles. This is due to the elemental tungsten on the surface of the diamond being transformed into tungsten carbide.

**Figure 4:**
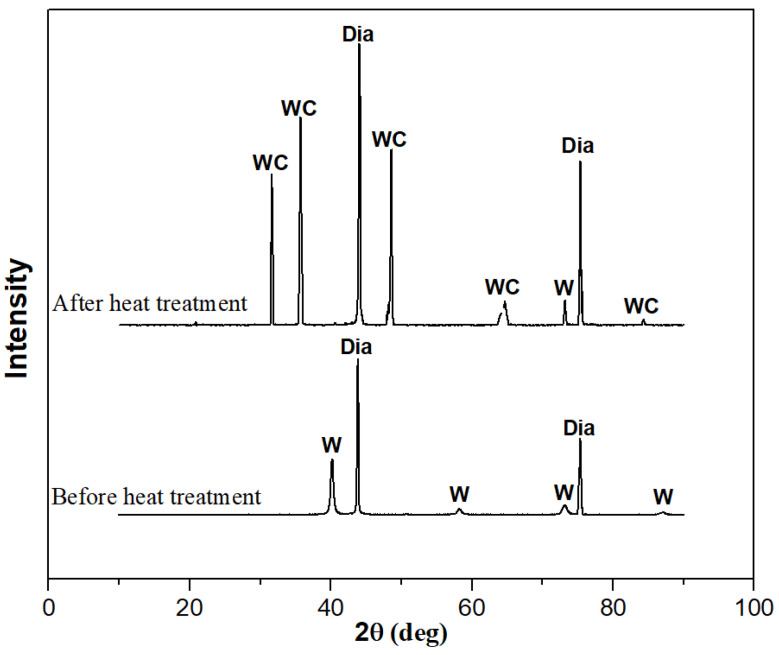
XRD patterns of diamond powder before and after heat treatment.

**Figure 5:**
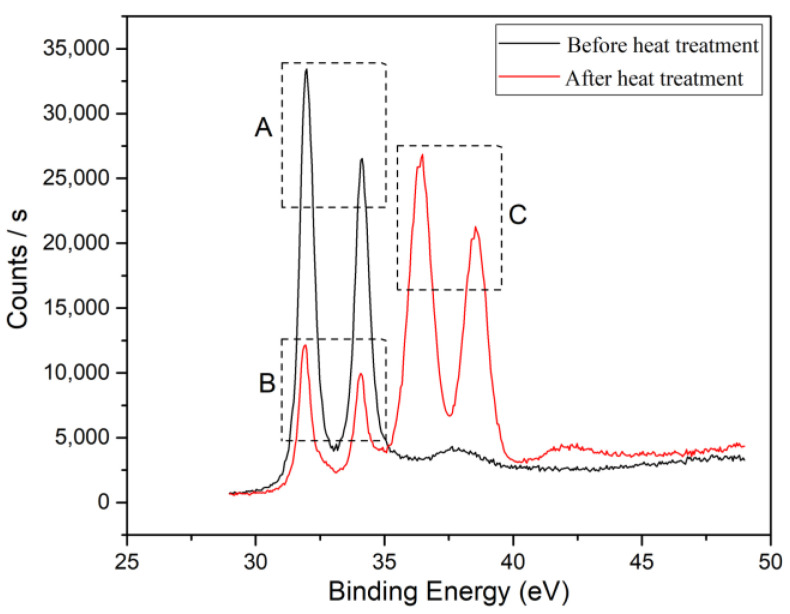
XPS patterns of diamond powder before and after heat treatment.

To confirm the formation of tungsten carbide on the surface of the diamond after heat treatment, XRD and XPS were used to detect the diamond powder before and after heat treatment, as shown in Figure 4 and Figure 5. From the XRD pattern in Figure 4, it can be seen that elemental tungsten undergoes a chemical reaction with diamond at a high temperature, forming tungsten carbide. Figure 5 shows the analysis results of the XPS. Ion etching was used to remove the 5~10 nm surface layer before the characterization of the sample. The pair of spectral peaks in box A represents the elemental tungsten before the heat treatment. The pair of spectral peaks in box B represents the residual elemental tungsten after the heat treatment. The pair of spectral peaks in the C box represents tungsten in the +4 valence state. The relative height of the spectral peak represents the content of tungsten in the corresponding valence state, indicating that most of the elemental tungsten in the coating has reacted with diamond to form tungsten carbide. Tungsten carbide will act as a glue between the diamond and aluminum alloy, enhancing the bonding strength at the interface and improving the overall thermal performance of the composite material.

### 3.2. Preparation of Aluminum Alloy/Diamond Composite Materials

The relationship between the laser power and spot temperature was systematically characterized before laser cladding. Excessively high point temperatures can lead to the graphitization of the diamond, while excessively low point temperatures can lead to the inadequate melting of the alloy matrix. The atmospheric environment has a significant impact on the thermal stability of diamond. The thermal stability of diamond varies greatly when heated in different atmospheres. Diamond begins graphitization in oxygen at approximately 660 °C. The graphitization temperature will increase to 800 °C in air. In vacuum or inert gas, the graphitization temperature of diamond will approach 1500 °C. The laser cladding experiment in this article was conducted in an argon atmosphere. The melting point of AlSi10Mg alloy is about 600 °C. Therefore, when preparing aluminum alloy/diamond composite materials by laser cladding, it is necessary to control the laser spot temperature within the range of 600~1500 °C.

**Figure 6:**
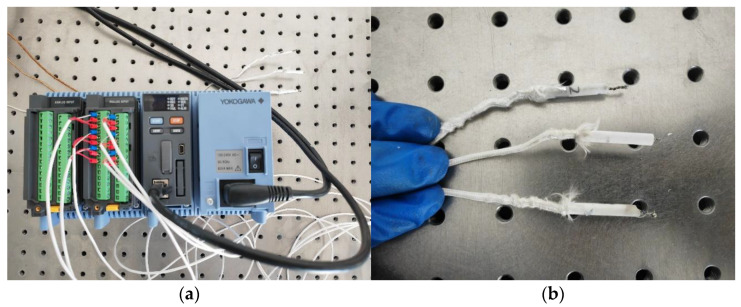
Testing experiment on the relationship between laser power and spot temperature: (**a**) thermometer; (**b**) thermocouple.

The Yokogawa thermometer shown in Figure 6 was used for the preliminary testing of the relationship between the laser power and spot temperature. The adjustment of the laser power to irradiate the thermocouple and the relationship between the laser power and the spot temperature obtained from the experiment are shown in Table 1. It can be seen that a laser spot at 907 °C to 1302 °C can be obtained in 0.2 s when the laser power is between 1000 W and 1400 W. In the laser cladding experiments, the heat dissipation of the aluminum alloy substrates also needs to be considered, so the laser power should be appropriately amplified based on the relationship between the laser power and spot temperature mentioned above.

**Figure 7:**
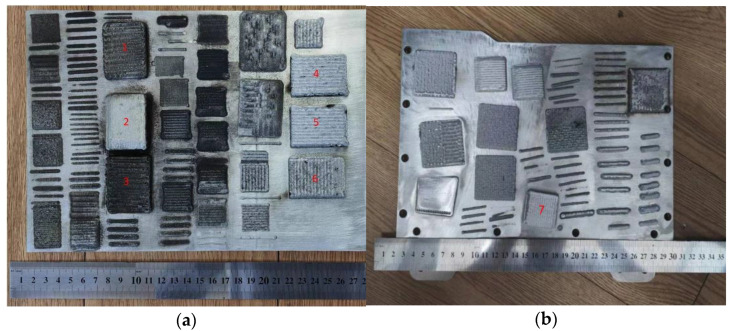
Samples prepared by laser cladding: (**a**) 1#~6# aluminum alloy/diamond composite materials; (**b**) 7# aluminum alloy.

The experimental design of laser cladding for the aluminum alloy/diamond composite materials is shown in Table 2. The aluminum alloy/diamond composite material and aluminum alloy prepared by laser cladding are shown in Figure 7.

From Figure 7a, it can be seen that composite materials with an apparent density and metallic texture can be prepared at laser powers of 1400 W and 1500 W. When the cladding power is 1600 W, the surface of the composite material appears to be a noticeably black gray color, which is caused by the graphitization of the diamond. High-quality aluminum alloys can also be prepared using a laser power of 1500 W, as shown in Figure 7b. The other key parameters are listed as follows: scanning speed of 10 mm/s, spot width of 3 mm, powder feeding rate of 13 g/min, powder feeding gas flow rate of 7 L/min, protective gas flow rate of 15 L/min, and overlap rate of 50%.

### 3.3. Microstructure of Aluminum Alloy/Diamond Composite Materials

**Figure 8:**
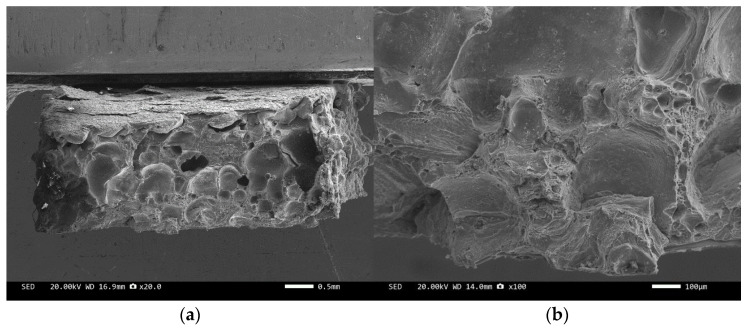
SEM image of sample 1#: (**a**) ×20, (**b**) ×100.

**Figure 9:**
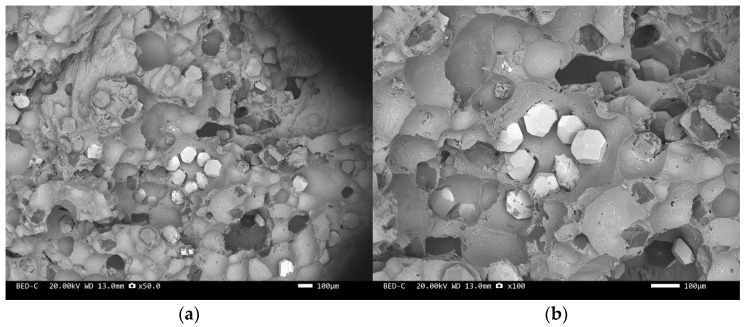
SEM image of sample 2#: (**a**) ×50, (**b**) ×100.

**Figure 10:**
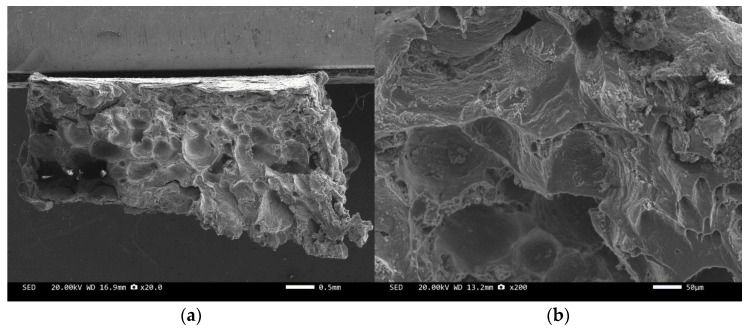
SEM image of sample 3#: (**a**) ×20, (**b**) ×200.

**Figure 11:**
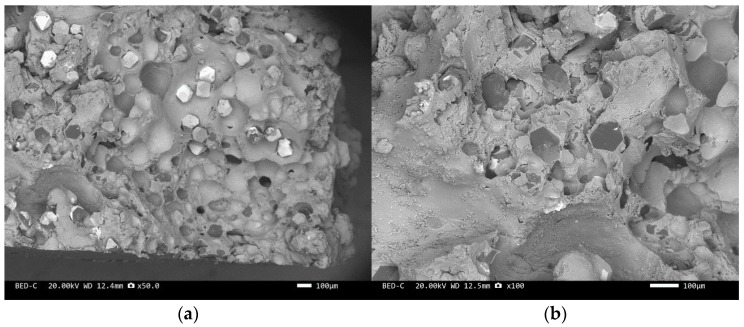
SEM image of sample 4#: (**a**) ×50, (**b**) ×100.

**Figure 12:**
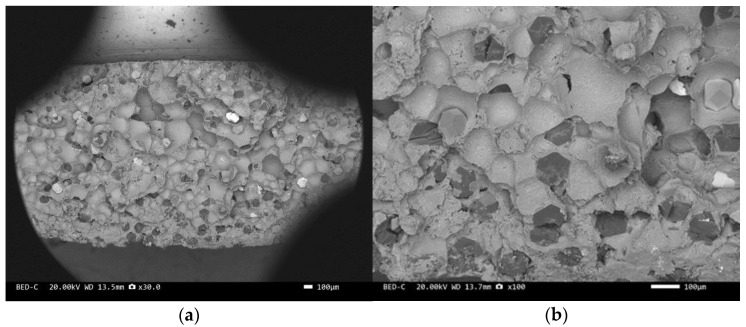
SEM image of sample 5#: (**a**) ×30, (**b**) ×100.

**Figure 13:**
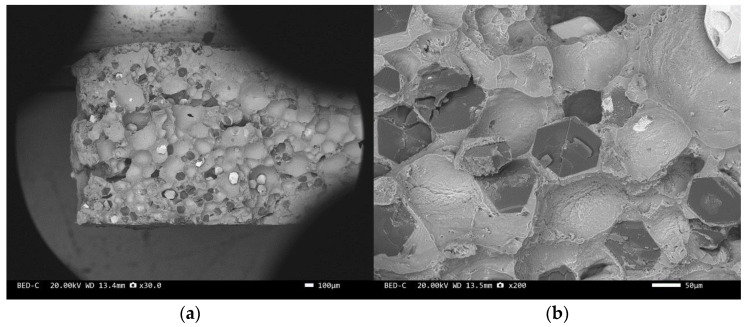
SEM image of sample 6#: (**a**) ×30, (**b**) ×200.

**Figure 14:**
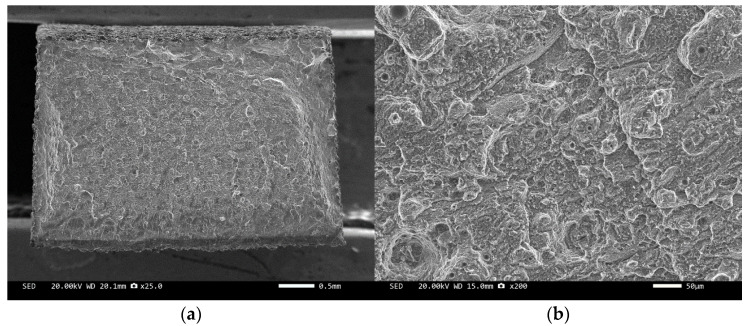
SEM image of sample 7#: (**a**) ×25, (**b**) ×500.

Three-point bending is used to break the composite material, and then scanning electron microscopy is used to observe the bonding between the diamond and aluminum alloy matrix, matrix defects, and diamond cleavage fracture. Figure 8, Figure 9, Figure 10, Figure 11, Figure 12, Figure 13 and Figure 14 correspond to the scanning electron microscope images of samples 1# to 7# after fracture, respectively.

Figure 8 and Figure 10 are scanning electron microscope images of the fracture surfaces of sample 1# and sample 3#. A large number of holes can be seen in the cross-section and no diamond particles were found. The diamond underwent a graphitization transformation at a laser power of 1600 W. The scanning electron microscope images of the fracture surfaces of sample 2# and sample 4# are shown in Figure 9 and Figure 11. The cross-section contains both intact and fractured diamonds. Although the diamond has not undergone graphitization, the bonding strength between the diamond and aluminum alloy substrate was insufficient at a laser power of 1400 W.

The scanning electron microscope images of the fracture surfaces of sample 5# and sample 6# are shown in Figure 12 and Figure 13. In the cross-section, only a relatively small amount of intact diamonds and a large amount of dissociated fractured diamonds are present. This indicates that the bonding strength between the diamond and aluminum alloy matrix is already very high, at a laser power of 1500 W. However, from the macrograph in Figure 7a, it can be seen that the surface of sample 6# appears light gray, indicating a possible slight transition from diamond to graphitization. The scanning electron microscope image of the fracture surface of the 7# aluminum alloy sample is shown in Figure 14. It can be seen that dense, less defect-free alloys can be prepared by using the same laser cladding process of the composites.

**Figure 15:**
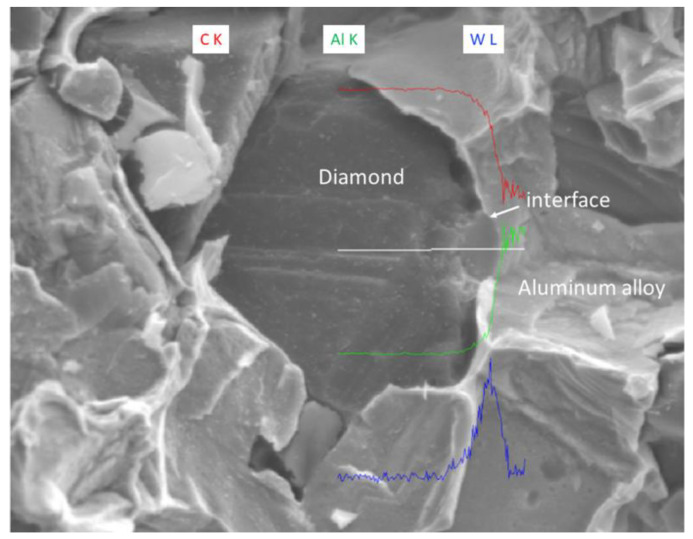
EDS analysis results of sample 6#.

**Figure 16:**
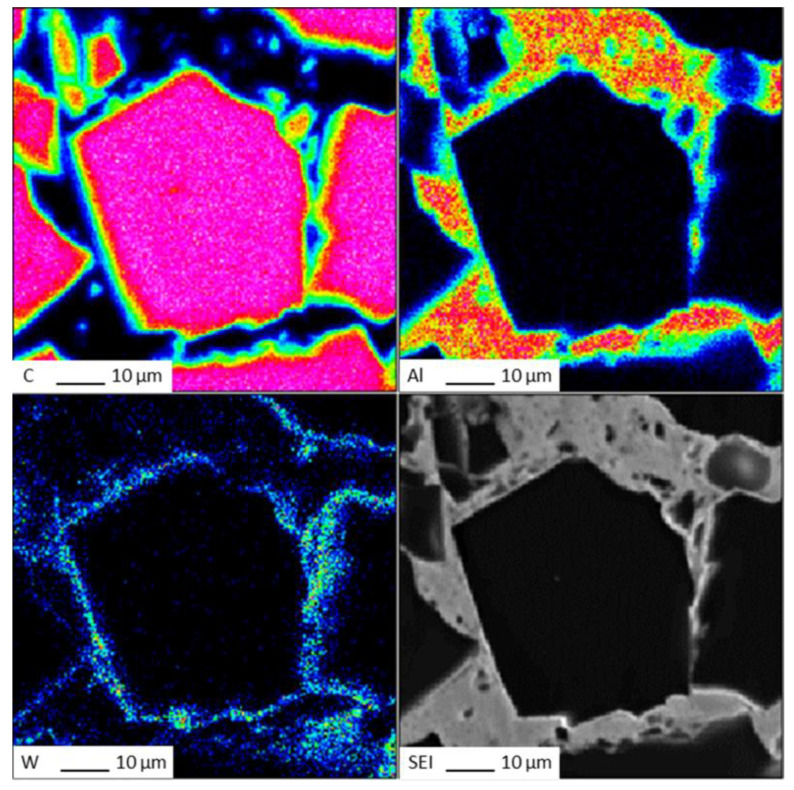
EPMA results for sample 6#.

The EDS (Energy Dispersive Spectroscopy) analysis results of sample 6# are shown in Figure 15, indicating that tungsten element did not migrate at the interface between the diamond and aluminum alloy. The EPMA (Electron Probe X-ray Microanalysis) results for sample #6 are shown in Figure 16, where tungsten element is uniformly distributed on the surface of the diamond. From the above experimental results, it can be seen that there is no interdiffusion of elements between the tungsten-plated diamond and aluminum alloy substrate during the laser cladding process. Aluminum alloy melts under the action of laser and tungsten-coated diamond is wrapped in liquid metal and then cooled to form an aluminum alloy diamond composite material.

### 3.4. Properties of Aluminum Alloy/Diamond Composite Materials

The mechanical and thermal properties of composite 5#, composite 6# and alloy 7# were tested with good forming quality.

The density of the material was measured by the weighted method and the drainage method, and the results are shown in Table 3. The densities of the aluminum matrix and diamond were calculated to be 2.70 g/cm^3^ and 3.51 g/cm^3^, respectively. The density of the material decreases with the increase in the diamond content, as shown in Table 3.

**Figure 17:**
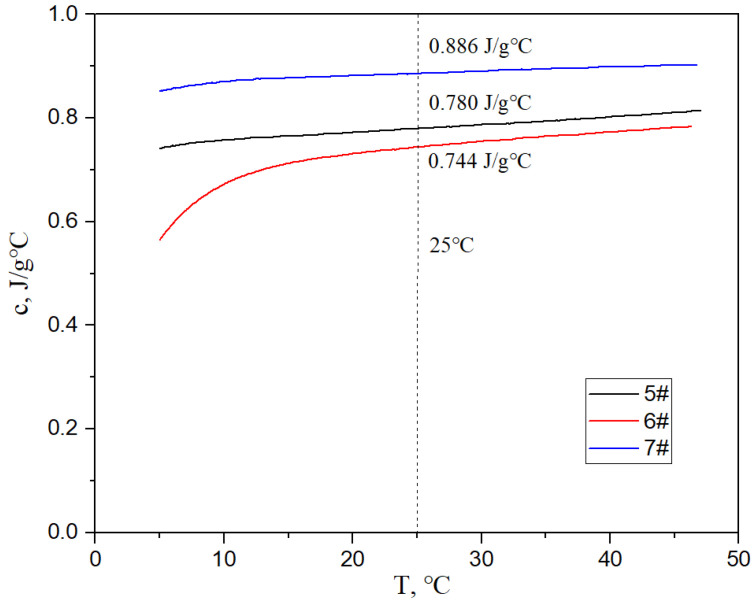
Specific heat curves of three materials.

**Figure 18:**
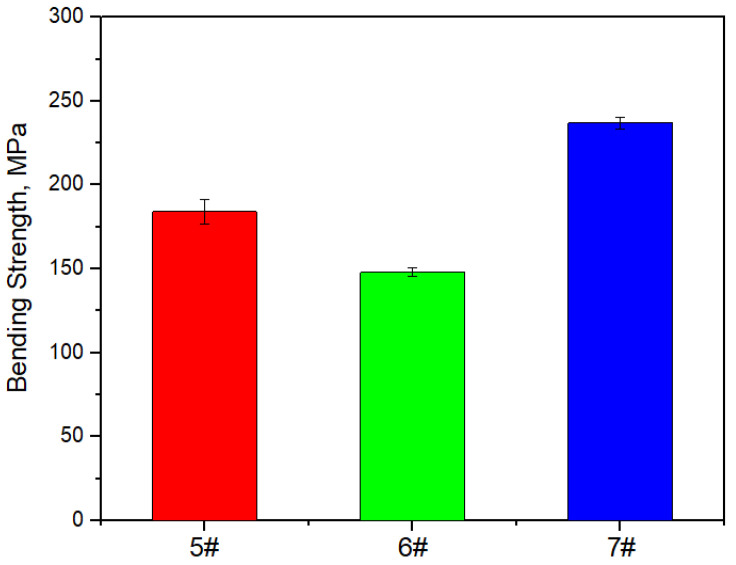
Bar chart of flexural strength of three materials.

**Figure 19:**
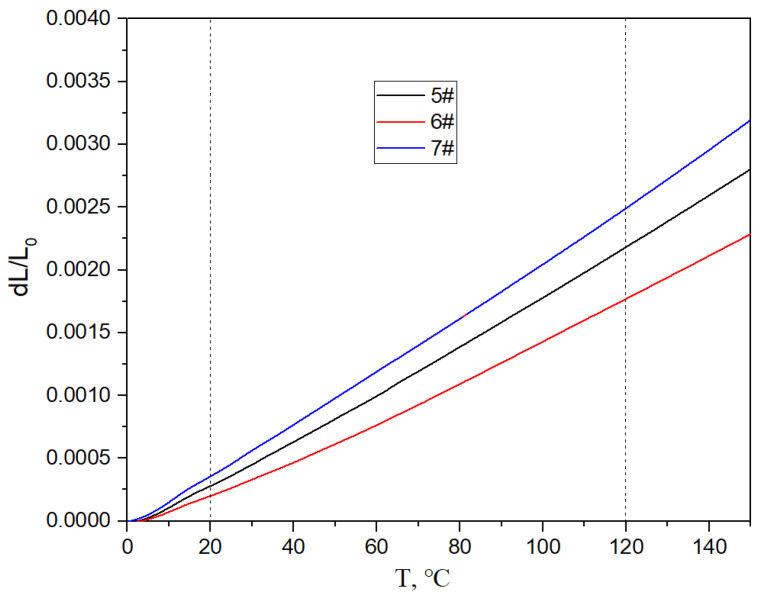
Linear thermal expansion curves of three materials.

Figure 17, Figure 18 and Figure 19 show the specific heat curves, flexural strength bar charts, and linear thermal expansion curves of three materials. The room temperature specific heats (c) of materials 5#, 6#, and 7# are 0.780 J/gk, 0.744 J/gk, and 0.886 J/gk, respectively, and the thermal diffusion coefficients (α) are 93.90 mm^2^/s, 83.73 mm^2^/s, and 57.60 mm^2^/s, respectively. The mathematical relationship between the thermal conductivity (λ) and thermal diffusivity (α) is λ = αρc, where ρ is the density and c is the specific heat. The room temperature thermal conductivities of materials 5#, 6#, and 7# were calculated to be 200.68 W/mK, 161.34 W/mK, and 135.75 W/mK, respectively, and the bending strengths are 184 MPa, 148 MPa, and 237 MPa, respectively. The average linear thermal expansion coefficients of materials 5#, 6#, and 7# within the temperature range of 20~120 °C (the operating temperature range of electronic devices) are 1.904 × 10^−5^/K, 1.575 × 10^−5^/K, and 2.140 × 10^−5^/K, respectively. The mechanical and thermal performance data of the three materials are shown in Table 4.

The thermal conductivity of the aluminum alloy diamond composite materials prepared by pressure infiltration and pressure casting is around 500 W/mK, and the volume fraction of the diamond in the composite material is about 60% [3,9]. The mechanical and thermal properties of aluminum alloy diamond composite materials prepared by laser cladding in this article are not as good as those prepared by traditional methods, but their advantage lies in the ability to prepare complex-shaped composite parts in a near net shape, and are almost not limited by size.

## 4. Discussion

A tungsten carbide layer was obtained on the surface of diamond through tungsten plating and vacuum heat treatment. The surface properties of the diamond have been altered, providing the possibility for the laser cladding preparation of aluminum alloy/diamond composite materials. A simple temperature measurement experiment was designed as shown in Figure 6 to calibrate the relationship between the laser power and instantaneous temperature of the spot. In the laser cladding experiments, it is necessary to control the “appropriate laser power” to achieve a spot temperature that can quickly melt aluminum alloy without causing the graphitization of diamonds.

From the macroscopic morphology of the sample shown in Figure 7, it can be seen that under the experimental conditions, the process of laser cladding with a power of 1400 W~1500 W, scanning speed of 10 mm/s, spot width of 3 mm, powder feeding rate of 13 g/min, powder feeding gas flow rate of 7 L/min, protective gas flow rate of 15 L/min, and overlap rate of 50%, respectively, can prepare aluminum alloy/diamond composite materials and aluminum alloys with intact surfaces.

When the diamond content is different, the “appropriate laser power” mentioned earlier is different. Due to different diamond contents and thermal properties of the materials, the temperature of the molten pool formed by the same power laser is not the same. The higher the diamond content, the higher the temperature of the material in the spot range. At the same laser power, there is a significant difference in the surface color between samples 5# and 6#, and the diamond in sample 6# undergoes slight graphitization.

On the fracture surface of the aluminum alloy/diamond composite materials, if a large amount of cleavage fracture occurs in the diamond, it means that the interface between the metal matrix and diamond is well bonded. The bonding between their micro interfaces directly affects the mechanical and thermal properties of the composite materials.

We have proposed that the more diamond cleavage fractures observed on the fracture surface, the better the mechanical and thermal properties of the composite material. From the microstructure photos of the composite materials in Figure 8, Figure 9, Figure 10, Figure 11, Figure 12 and Figure 13, it can be seen that the bonding between the diamond and aluminum alloy is the best in sample 5, followed by that in sample 6. At a laser cladding power of 1500 W, the bonding of the resulting composite micro interfaces was also better than that at 1400 W.

The following phenomena were discovered during the testing of the mechanical and thermal properties of the material. With the increase in the diamond content, the density, specific heat, mechanical properties, and average linear thermal expansion coefficient of the material gradually decrease, and the thermal conductivity of the material first increases and then decreases.

The lower the porosity of the material, the better its mechanical and thermal properties should be. The higher the diamond content, the more difficult it is to form a composite material and the higher the porosity. The specific heat of composite materials is only affected by their components. The higher the diamond content in the composite (0.470 J/gK for diamond and 0.886 J/gK for AlSi10Mg alloy), the lower the overall specific heat of the composite.

**Figure 20:**
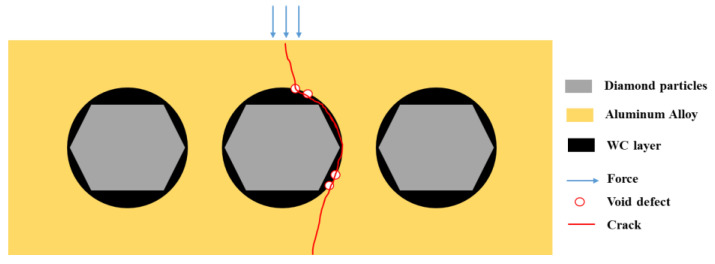
The schematic diagram of bending fracture of composite materials.

The mechanical properties of the material exhibit a significant “cask effect”, where the internal pore defects and micro interfaces of the composite material are the “short boards of the bucket”. And pore defects often appear at the micro interface, so the mechanical properties of the composite materials significantly decrease with the increase in the diamond content. The schematic diagram of the bending fracture of the composite materials is shown in Figure 20. Cracks often initiate at the defect of the interface and propagate along the interface, leading to material fracture, which greatly weakens the bending strength of the material.

The thermal conductivity and thermal expansion performance of the materials are the result of the combined effect of the diamond content and interface. Diamond particles are excellent conductors of heat, but the thermal conductivity of the micro interface between the diamond and aluminum alloy is weaker than that of the diamond and aluminum alloy. The overall thermal conductivity of composite materials is determined by the average thermal conductivity along the heat transfer path.

**Figure 21:**
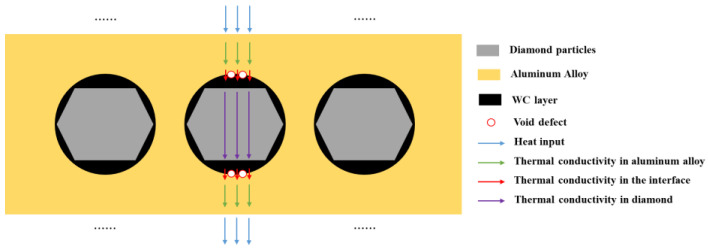
The schematic diagram of thermal conductivity of composite materials.

When the number of diamond particles increases, it has a positive impact on the thermal conductivity of the material. When the number of interfaces increases, it has a negative impact on the thermal conductivity of the material, especially for interfaces with more defects, where the decrease in thermal conductivity is more significant. The increase in the diamond content leads to an increase in defects in the composite material, which introduces a large number of defects at the interface, resulting in a sharp decrease in the thermal conductivity of the material. The schematic diagram of the thermal conductivity of the composite materials is shown in Figure 21, and the thermal conductivity of each part in the thermal conduction chain jointly determines the overall thermal conductivity of the composite material.

For the thermal expansion performance, the higher the content of diamond with a lower thermal expansion coefficient, the lower the overall thermal expansion coefficient of the material. And the stronger the micro interface bonding between the diamond and aluminum alloy, the greater the binding force of the diamond on the aluminum alloy, and the lower the expansion coefficient of the material. For the material system in this article, the content of diamond is clearly the main factor. Therefore, even though the micro interfacial bonding of sample 6# is not very good, the coefficient of expansion is low due to the high diamond content.

## 5. Conclusions

In summary, diamond/aluminum alloy composites were prepared using laser cladding, the microstructure and thermal properties of the composites were characterized, and then the reasons for the changes in properties were analyzed. The main conclusions are as follows.

1. Tungsten is deposited on the surface of diamond by magnetron sputtering, and a uniform tungsten carbide layer can be obtained on the diamond surface by short-term vacuum heat treatment at 1150 °C for 15 min.
2. Under the conditions of a power of 1500 W, scanning speed of 10 mm/s, spot width of 3 mm, powder feeding speed of 13 g/min, powder feeding gas flow rate of 7 L/min, protective gas flow rate of 15 L/min, and overlap rate of 50%, high-density aluminum alloy/diamond composites and aluminum alloys can be prepared through the laser cladding process.
3. With the increase in the diamond content, the density, specific heat, mechanical properties, and average linear thermal expansion coefficient of the material gradually decrease, and the thermal conductivity of the material first increases and then decreases.
4. When the diamond content is 15% and the aluminum alloy content is 85%, the thermal conductivity of the composite material reaches 147% of the aluminum alloy matrix, the bending strength decreases to 77% of the aluminum alloy matrix, and the thermal expansion coefficient decreases to 89% of the aluminum alloy matrix.

## Figures and Tables

**Table 1:** Experimental results of testing the relationship between laser power and spot temperature.

No.	Time, s	Power, W	Temperature, °C
1	0.2	200	392
2	0.2	500	451
3	0.2	750	496
4	0.2	1000	907
5	0.2	1200	940
6	0.2	1400	1302
7	0.2	1600	Burn down

**Table 2:** Process parameters of laser cladding experiment.

Name	Component	Power, W
1#	70% Aluminum alloy + 30% Dia	1600
2#	70% Aluminum alloy + 30% Dia	1400
3#	85% Aluminum alloy + 15% Dia	1600
4#	85% Aluminum alloy + 15% Dia	1400
5#	85% Aluminum alloy + 15% Dia	1500
6#	70% Aluminum alloy + 30% Dia	1500
7#	100% Aluminum alloy	1500

**Table 3:** Calculation and measured density of materials.

Name	Weighted Density, g/cm^3^	Drainage Method			Porosity, %
		Mass in Air, g	Mass in Water, g	Measured Density, g/cm^3^	
5#	2.86	0.5842	0.3710	2.74	4.20%
6#	2.94	0.5705	0.3502	2.59	11.90%
7#	2.70	1.2916	0.8060	2.66	1.48%

**Table 4:** The mechanical and thermal performance data of the three materials.

Name	ρ, g/cm^3^	c, J/gk	α, mm^2^/s	λ, W/mK	Bending Strength, MPa	Linear Coefficient of Thermal Expansion, 1/K
5#	2.74	0.780	93.90	200.68	184	1.904 × 10^−5^
6#	2.44	0.744	83.73	161.34	148	1.575 × 10^−5^
7#	2.66	0.886	57.60	135.75	237	2.140 × 10^−5^

## Data Availability

Data is unavailable due to privacy restrictions.
